# Baited traps as flawed proxies for carcass colonization

**DOI:** 10.1038/s41598-025-90522-1

**Published:** 2025-02-20

**Authors:** Lena Lutz, Jens Amendt, Gaétan Moreau

**Affiliations:** 1 Institute of Legal Medicine, University Hospital Frankfurt am Main, Goethe-University, Kennedyallee 104, D-60596 Frankfurt am Main, Germany; 2 Département de biologie, Université de Moncton, New Brunswick, E1A 3E9 Canada

**Keywords:** Surrogates, Random forest, Blow flies, Oviposition activity, Ecological modelling, Ecology, Behavioural ecology

## Abstract

In both fundamental and applied sciences, the use of surrogates to measure phenomena that are challenging to study directly is a common practice. However, this requires validating the appropriateness of the surrogates. This study examines if traps, used to measure flight activity of necrophagous flies, can serve as effective surrogates for predicting oviposition on whole carcasses, a topic still under debate in forensic science. We used three sets, a calibration and validation subsets comprising monitoring data of the flight activity of four necrophagous blow fly species, and a test set comprising the oviposition activity of these species on carcasses. Each set also included measurements of abiotic parameters. Using Random Forest for each species, we quantitatively and qualitatively modeled flight activity as a function of abiotic parameters and validated these models. However, when we examined the extent to which flight activity predicted oviposition on carcasses, the models performed poorly, only explaining a fraction of the variance. As the first study making use of small baited trap data to model oviposition on animal carcasses, this study presents mixed results that suggests that traps, despite their utility in addressing various forensic entomology questions, currently appear to be unreliable proxies for predicting carcass colonization.

## Introduction

Comprehending and predicting the oviposition activity and behavior of insects is paramount for their management in various domains such as pest control^1^, vector entomology^2^, conservation biology^3^ and forensic entomology^4^. In the latter field, accurately determining the precise timing of insect oviposition on cadavers is particularly important, as it establishes a timeline that is key evidence in forensic investigations^4,5^. This requires describing, understanding and predicting the insect fauna associated with human decomposition, including their succession and interaction on a cadaver, as well as their influence on carcass decay^6–8^. Since the use of human carcasses for research purposes is highly restricted and mainly takes place at human taphonomy facilities^9,10^, forensic entomological research often relies on human carcass surrogates^11^. Typically, for decomposition, succession and case studies, the surrogates for human carcasses are animal models, with pigs being the most frequently used^11^ along with other large or small vertebrates such as mice, rats or rabbits^12–14^. Meanwhile, studies that focus more on describing the seasonal activity of necrophagous insects tend to rely on baited traps^15–18^.

Surrogacy is not limited to forensic entomological research as it is widely used and discussed in medicine, pharmacology and ecology^19^, and has been a useful tool for linking fundamental and applied research^20^. The definition of a surrogate is “a proxy measure for an attribute of true interest that is too difficult or costly to measure directly”^20^ and which is used to make inference about the trend or status of such an attribute^21,22^. To date, little work has been done on the predictive capacity of a surrogate or the circumstances under which it works in forensic entomology. Whether and to what extent traps and animal models can serve as a substitute for a whole carcass or human body remains open to debate^11,23^. However, the emerging consensus is that both methods can provide useful baseline data on the biodiversity of a specific region^24–26^, the flight activity of first colonizers such as blow flies^15,27,28^ or successional patterns of necrophagous species on a carcass, caution must be exercised when extrapolating data from such surrogates to forensic cases^23^. Many studies rely on small baited traps, but few use the resulting data as a basis for prediction models^15,29–31^, and no study has verified whether monitoring data from traps can predict oviposition activity on cadavers. This is a fundamental question with profound implications for both the theoretical frameworks and the practice of forensic entomology. Forensic entomology establishes a minimum PMI (PMI_min_), the time between the initial colonization of a corpse by insects and its discovery. This colonization may or may not occur immediately after death. Understanding the factors that can cause a delay and discussing them when assessing the case is very important to the investigation. One of the prerequisites is a thorough knowledge of the behavior of insects in searching, finding and colonizing the body. In the current study, we address whether the sampling of adult necrophagous insects can be used to predict oviposition and initial colonization in animal models.

A method of assessing the validity of surrogacy is to develop a predictive model based on calibration data for the surrogate, validate this model, and check how well it predicts the attribute of true interest. To do this, one of the best approaches involves the use of computationally intensive and primarily data-driven algorithms known as “machine learning” such as the random forest^32^. Herein, to determine how well small baited traps inform us about the oviposition behavior of necrophagous organisms on carcasses, we developed models based on abiotic parameters predicting which of the most forensically important blow fly species are active in the adult stage throughout the year. These models were validated with a subset on adult blow flies, and then tested to determine whether they explain oviposition on carcasses. We developed both quantitative models to examine whether the abundance of adult blow flies in the traps is related to the abundance of juveniles on a carcass, and qualitative (i.e., presence-absence) models accounting only for the presence/absence of adults and juveniles. Considering that traps and carcasses differ in terms of tissue exposed, odor emitted and susceptibility to colonization^23^, not to mention possible interspecific competition at the time of oviposition, we predicted that the composition of blow fly communities would differ between traps and carcasses. However, the primary aim was not simply to highlight the difference, but to quantify it, thereby providing a clearer understanding of how the quality of inference derived from these traps is impacted. Similarly, as quantitative models examine both presence and abundance, whereas qualitative models only examine presence, we expected qualitative models to perform better than quantitative ones.

## Results

### Abundance data

**Fig. 1:**
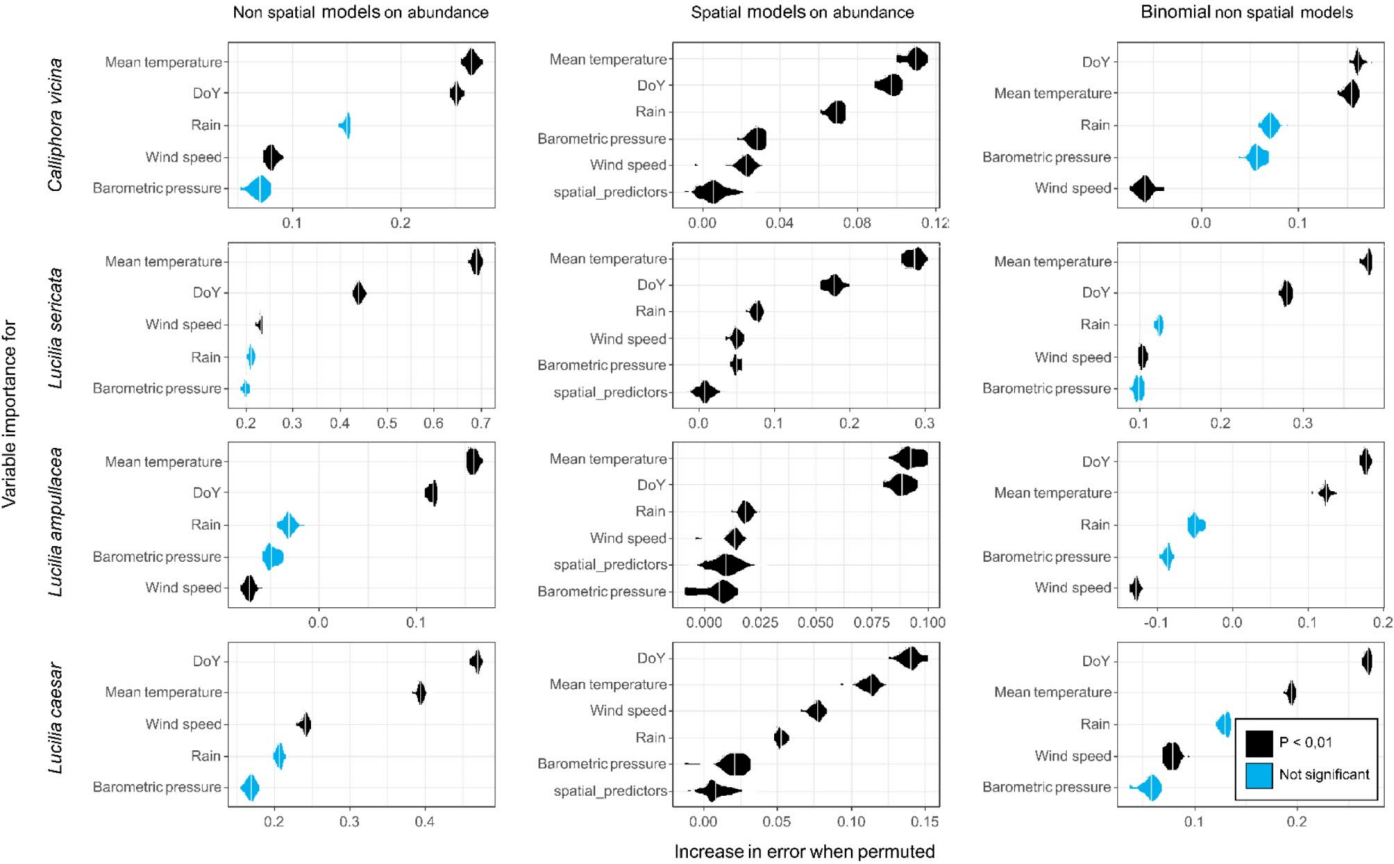
Violin plots of predictor importance in quantitative non-spatial, quantitative spatial, and qualitative non-spatial random forests for 30 runs of each model for each species. The probability that a predictor is in more nodes than expected by chance is indicated by the color of the violin. The parameters are mean day temperature (°C), rain in mm, wind speed (km h ^− 1^), barometric pressure (hPa), and day of the year (DoY).

**Fig. 2:**
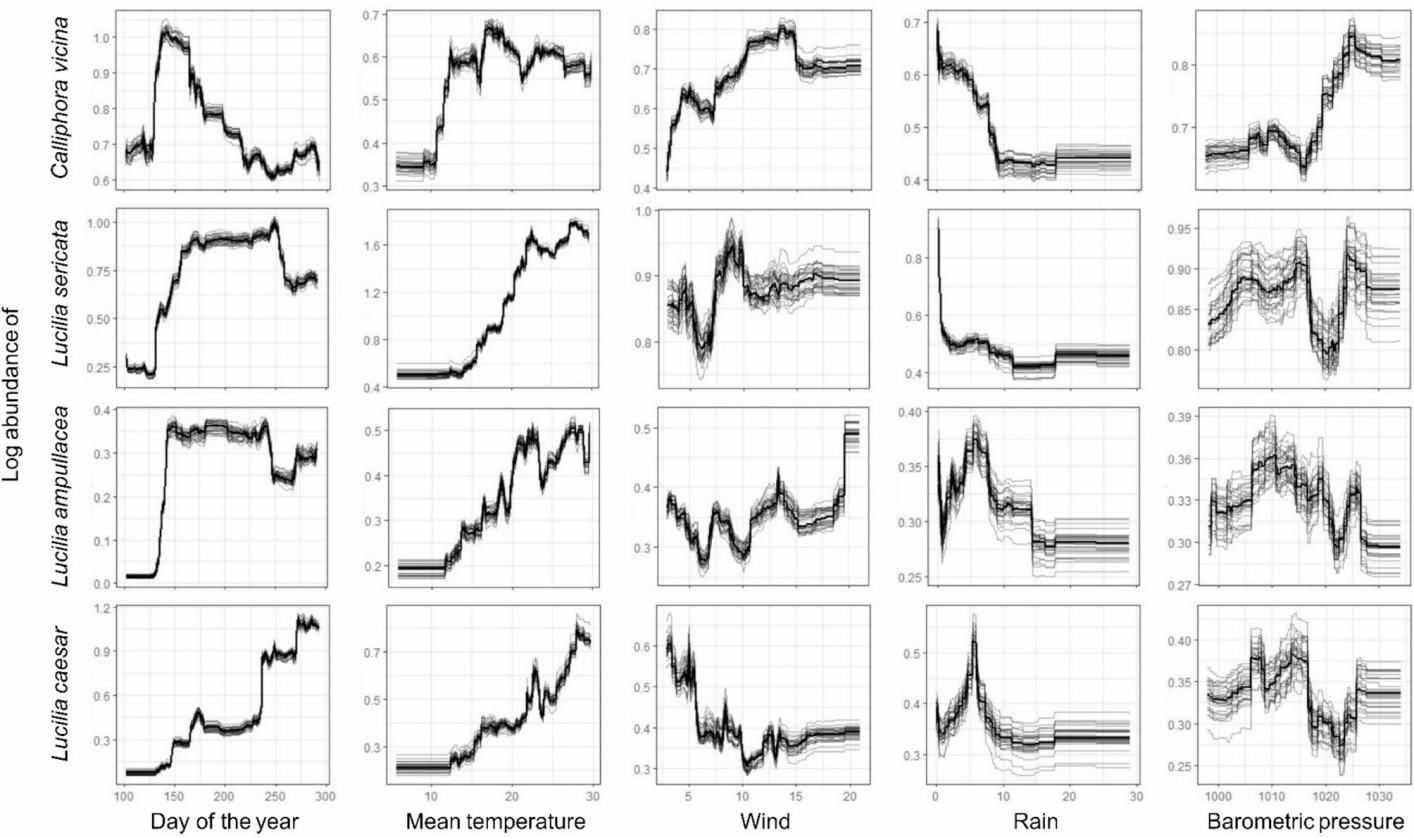
Respond curves of the five predictors of non-spatial random forests on abundance data. Each of the grey lines on a figure represents one of 30 repeated executions of each model and the black line represents the average response. The values of a predictor are estimated by setting the other predictors to their median value.

**Fig. 3:**
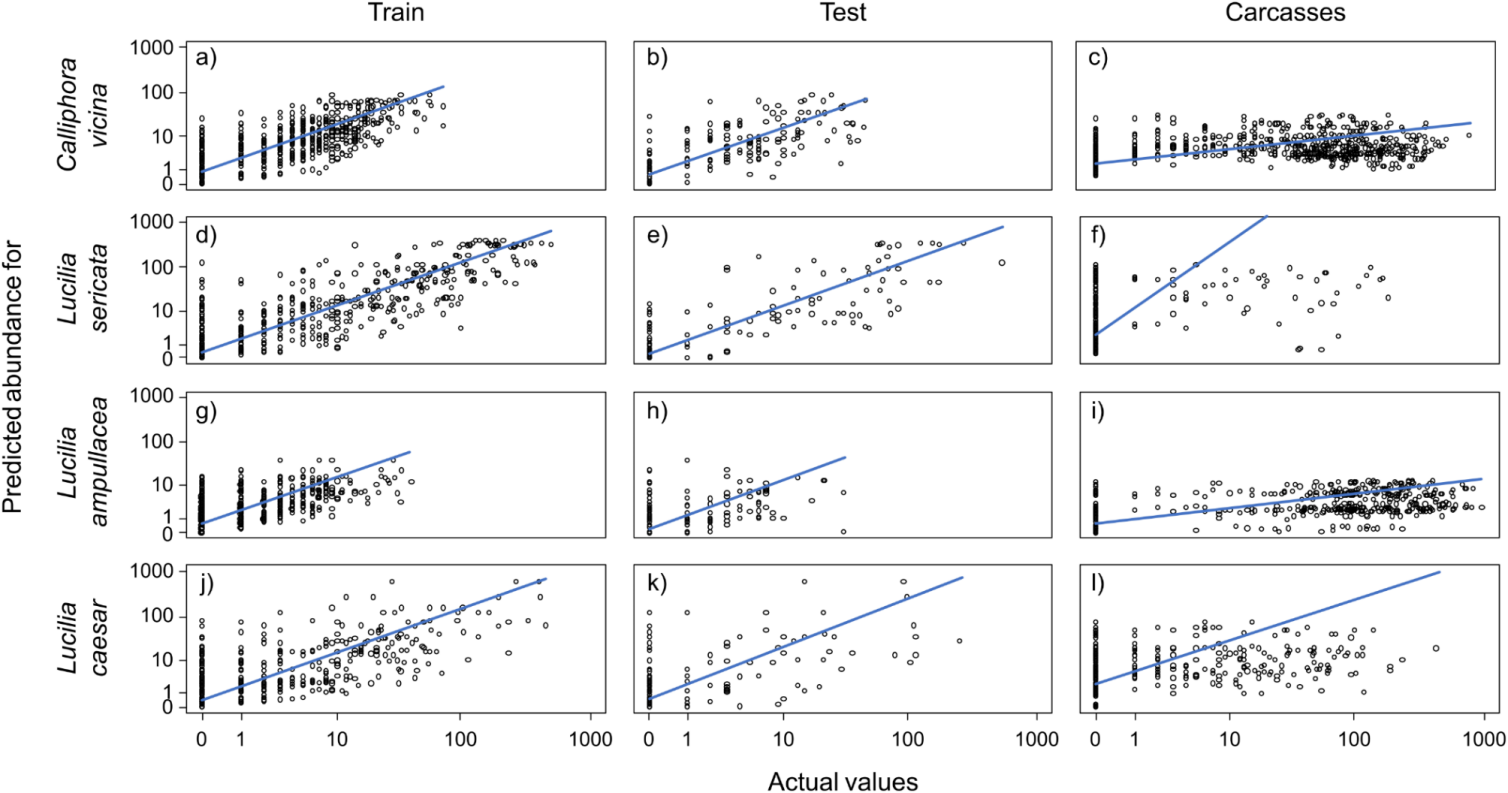
Estimated values of abundance predicted by the random forest models as a function of actual measurements for the calibration subset (a, d, g, j), validation subset (b, e, h, k) and carcass (c, f, i, l) dataset. The lines are the estimated relationships between the predicted and actual values obtained using Standardized Major Axis regression.

Quantitative RF models were developed for four blow fly species using a calibration subset to identify the effect of the environmental predictors on blow fly abundance in baited traps. The models explained 39–75% of the variance in blow fly abundance, the most influential predictors always being the day of year and the mean temperature (Table 1; Figs. 1 and 2). *Calliphora vicina* was positively influenced by a mean day temperature over 10 °C up to 20 °C and showed a bimodal seasonal activity with peaks in spring and autumn. Wind and barometric pressure had also a positive effect on the flight activity whereas precipitation over 10 mm negatively influenced the flight of this species. *Lucilia sericata* showed similar trends, being positively influenced by a mean temperature over 15 °C and having a flight activity restricted to summer. Precipitation had a strong negative impact on its flight activity, which ceased even with very small amounts of rain. The barometric pressure influenced *L. sericata* positively up to 1015 hPa, whereas higher values had a strong negative effect. No clear effect of wind speed was apparent. *Lucilia ampullacea* was positively influenced by a mean temperature over 10 °C, albeit with a slow increase in activity as the temperature rose. This species was active throughout the entire spring, summer, and autumn without a clear preference for one season over another. Precipitation had an overall negative effect on its flight activity, whereas barometric pressure negatively influenced activity above 1020 hPa. Compared to other species, *L. ampullacea* appears to be less affected by the abiotic parameters of the study. Temperatures above 15 °C had an overall positive effect on *L. caesar* flight activity, which slowly increased throughout the year to reach its peak highest abundance in autumn. Unlike all other species, wind had a strong negative impact on flight activity, whereas precipitation and barometric pressure had positive effects, but only within a narrow range of parameters. The models were relatively powerful in predicting blow fly abundance in the calibration subset (Table 1; Fig. 3a, d, g, j). When these models were applied to the validation subset, the fit and the quality of predictions declined to 20‒68% of the variance explained (Table 1; Fig. 3b, e, h, k). Positive autocorrelation being detected in all RFs, spatial RFs were developed to assess the influence of spatial effects on flight activity. The spatial models explained a smaller proportion of the variance, identified the same predictors as important, and all indicated that spatial predictors had little influence on blow fly abundance (Table 1; Fig. 1). We infer that spatial models are not needed here.

**Table 1:** Summary of random forests carried out to predict the abundance or presence/absence of four blow fly species recovered from baited traps (calibration and validation) or from mouse carcasses in urban areas of Frankfurt, Germany.

Species	Model	Dependent	*r*^2^ (% variance explained)				Prediction accuracy (%)		
			Calibration	Validation	Carcasses		Calibration	Validation	Carcasses
*Calliphora vicina*	non spatial	abundance	0.53	0.40	0.27		-	-	-
	spatial	abundance	0.27	-	-		-	-	-
	non spatial	presence	0.41	-	-		0.79	0.72	0.73
*Lucilia sericata*	non spatial	abundance	0.75	0.68	0.07		-	-	-
	spatial	abundance	0.39	-	-		-	-	-
	non spatial	presence	0.65	-	-		0.87	0.84	0.68
*Lucilia ampullacea*	non spatial	abundance	0.39	0.20	0.38		-	-	-
	spatial	abundance	0.32	-	-		-	-	-
	non spatial	presence	0.38	-	-		0.76	0.68	0.80
*Lucilia caesar*	non spatial	abundance	0.58	0.38	0.12		-	-	-
	spatial	abundance	0.25	-	-		-	-	-
	non spatial	presence	0.50	-	-		0.81	0.66	0.75
Whole complex	non spatial	presence	-	-	-		-	-	0.46

The quantitative RF models were applied to the mouse carcass dataset to determine how well baited trap data can predict blow fly oviposition on carcasses. This time, the variance explained dropped to 7–38% (Table 1). A graphical representation of the RF predictions shows that the models developed for *L. sericata* and *L. caesar* were ineffective in part because these species were rarely documented on mouse carcasses (Fig. 3f, l). The models perform better for *C. vicina* and *L. ampullacea*, but in both cases they predict values far below documented abundance (Fig. 3c, i).

### Presence-absence data

To determine whether a model that does not include abundance might perform better than the quantitative model described above, qualitative RF models were developed for each of the four species using the calibration subset to identify the effect of the predictors on blow fly occurrence (i.e., presence/absence) in baited traps. The models explained 41–65% of the variance in blow fly occurrence (Table 1). As with RF models on abundance, the day of year and the mean temperature were by far the best predictors of blow fly occurrence (Fig. 1). The prediction accuracy of these models varied between 76 and 87%, which means that the models were right in predicting occurrences 76 to 87% of the time (Table 1). When the qualitative models were applied to the validation subset, the quality of predictions declined to 66–84% (Table 1).

The qualitative RF models were applied to the carcass dataset to determine how well baited trap data can predict blow fly occurrence (or absence) on carcasses. In this case, the models were right in predicting the occurrence 68 to 80% of the time (Table 1), which is similar to the validation subset. However, an examination of the frequency of true and false predictions indicates that the model was effective in predicting the absence of *C. vicina* but performed poorly when predicting the presence of this species, as it was correct 53.8% of the time, which is only slightly better than the result obtained by flipping a coin (Table 2). The opposite situation was observed with *L. sericata*, the model being efficient in predicting presence but being accurate only 66.4% of the time when trying to predict absence (Table 2). The models of *L. ampullacea* and *L. caesar* occurrence were more accurate (Table 2).

**Table 2:** Univariate test using 1140 carcasses of the predictive power of qualitative random forest developed from baited traps.

Species	Absence correctly predicted	Absence incorrectly estimated as presence	Presence correctly predicted	Presence incorrectly estimated as absence
*Calliphora vicina*	536	56	295	253
*Lucilia sericata*	722	366	48	4
*Lucilia ampullacea*	620	128	290	102
*Lucilia caesar*	696	267	154	23

When the predictions of all four qualitative models were combined to determine the overall ability of the baited trap data to predict the blow fly community, the result was poor (Table 3). Although the model predicted 4 out of 4 species 46.3% of the time, this reflects the fact that the models had no difficulty predicting the absence of all 4 flies during the coldest times of the year, which accounted for 39.6% of the accurate predictions (Table 3). However, when one, two, or three species were present, the models performed poorly (Table 3). On the positive side, the model was quite successful in predicting the presence of all four species when they were all present at the same time (Table 3).

**Table 3:** Multivariate test using 1140 mouse carcasses of the predictive power of qualitative random forest developed from baited traps.

Number of co-occurring species	4/4 accurate predictions	3/4 accurate predictions	2/4 accurate predictions	1/4 accurate prediction	0/4 accurate prediction
0	452	50	11	18	7
1	16	118	31	33	19
2	2	32	130	61	4
3	2	74	46	8	0
4	18	7	1	0	0

## Discussion

The findings of this study demonstrate that quantitative or qualitative models based on abiotic parameters, which accurately describe adult blow fly dynamics, fall short in explaining oviposition dynamics and community composition on animal carcasses. In essence, adult trapping data and abiotic factors alone cannot adequately account for the abundance or presence/absence of larvae on carcasses. These results align with our prediction that small bait traps and mouse carcasses would host distinct blow fly communities. Additionally, we found that presence/absence models performed slightly better than abundance models in predicting blow fly activity for each species, supporting our expectation that qualitative models would outperform quantitative ones.

In a first step, we built models to explain the dependence of blow fly activity on abiotic parameters, a question of great importance in forensic entomology^15,26,33^. Often, general models for blow flies are developed^18,34^, including behavioral generalizations across this insect family, which fail to recognize the species-specific responses to thermal preferences or adaptations to abiotic parameters. While there are general dependencies that apply to all insects, each species, or even different populations of the same species, can develop specific adaptations, thus responding differently to the same parameter at a finer scale than another species^4,35^. This was also evident in the present study: although mean daily temperature and day of the year were the most important factors for all species, the variance explained by the selected parameters ranged between 40 and 75% for the different species. *Lucilia sericata* was by far the most abundant species in the calibration and validation subsets, and 75% of the variance of its activity was explained by the selected abiotic parameters, with mean temperature alone being the most important one. This result is consistent with previous studies, which defined this species as a high summer, sun-loving species whose activity is largely determined by temperature and season^33,36–38^. The dominance of this species in summer is also mirrored in the data of colonization of human corpses in Germany^39^, as well as in other European countries^40^. The combination of their behavior of arriving first at a carcass to reduce competition^41^ with the need for fresh carrion as protein source for the maturation of their ovaries^42–44^, results in a high number of females of different reproductive status in the traps. Overall, the flight activity of this species can be predicted well on the basis of climatic parameters. All other species in the present study seem to be less influenced by the selected abiotic parameters. For *C. vicina*, a year-round active species^4^, only ~ 50% of the activity could be explained, possibly because the calibration and validation subsets were limited to late spring to early autumn and thus did not cover the full activity of this species. This highlights the need for monitoring studies that are replicated for at least two years. *Lucilia ampullacea* is still poorly understood, although it frequently occurs on human carcasses^39^ and in baited traps^15,45^. At present, we simply do not have a good understanding of this species, its biology, and the driver for its flight and oviposition activity, which the results have highlighted. The model herein could only explain 39% of the variance in the data, suggesting that the dynamics of this species are driven by other abiotic or biotic factors. We emphasize that for *L. ampullacea* to eventually be used in forensic entomology, future basic research should focus on the ecology and life histories of this species.

One aspect that was probably not sufficiently addressed in earlier studies pertains to the value of baited traps as a carcass substitute and relates to oviposition ecology. Baited traps capture all individuals, irrespective of their ability to compete and decision-making once attracted to necromass. This implies that the number of specimens caught in baited traps will not be representative of those that would be found on carcasses in the same environment, since, unlike the situation on carcasses, the flies cannot exit the trap, even if the necromass is unsuitable for oviposition. On our carcasses, we did not monitor whether a species just landed on the carcass and left, but only which species had laid eggs that subsequently developed successfully. However, when approaching a carcass or landing on it, gravid females make their choices based in a range of biotic factors, including the abundance of competitors or predators^41,46,47^ and probably other factors that are not relevant for flight activity or have not yet been uncovered by research. As a result, some species abundantly sampled as adults near carcasses will often be poorly represented in rearing from these same carcasses, and vice versa^48^. The prediction of oviposition by *L. sericata* in the present study exemplifies this problem. We found a major discrepancy between the strength of this model for predicting adult activity and its actual capacity to predict colonization of mouse carcasses. This is explained by the predominance of this species in baited traps and, correspondingly, by a low number of larvae on mouse carcasses. One possible explanation for this is *L. sericata* being a poor competitor^49–52^. As such, it avoids or reduces competition by utilizing carcasses as soon as possible^53^, laying single egg clutches with high numbers of eggs^54^, having shorter developmental times and needing less food compared to other blow flies^41^. Therefore, the shortage and size of carcasses limits the increase of its population by reducing the emergence of adult flies^53,55^. Due to the habit of *L. sericata* of laying more eggs on a carcass than the tissue can support, there can be a sharp decline in their population during larval development, so it is the development after oviposition that is reduced rather than oviposition itself, as seen from the ratio of eggs laid to adults hatched^56^. On small carcasses, such as the one in our study, this behavior leads to an almost complete reduction in the population, but on human carcasses, the same strategy is very successful because food is less of a limiting factor. To support this hypothesis, however, will require similar work but using larger vertebrates or human carcasses, which is the outlook for future research.

Comparisons between adults and eggs/larvae found on a carcass in terms of presence/absence will generally be good. Yet, this study teaches us that a model using local surveys of adults and abiotic factors poorly predicts the community of blow flies on carcasses in the same environment, leading us to suggest that biotic factors are involved. We therefore emphasize that a better understanding of the influence of biotic factors on the behavior of gravid adults in the vicinity of carcasses could prove essential to improve our understanding and modeling of blow fly oviposition.

This study effectively refutes the notion that with current methodologies, small bait traps serve as an appropriate surrogate for animal carcasses regarding quantitative presence data of forensically important blow fly species. However, we have no desire to throw the baby out with the bathwater. Baited traps remain a valuable tool in forensic entomology, offering critical data on the diversity, abundance, activity and distribution of necrophagous species. Additionally, they provide insights into how these factors are influenced by abiotic parameters^15,57^. In addition, the low cost and ease of construction and installation of these systems allows for large scale replication, which is critical to the robustness of research data and resulting models, and is often overlooked in field studies using animal cadavers^58^. Furthermore, for the attraction of the species of interest, various types of baits can be used to allow the sampling of a particular age, sex or embryogenic status of necrophagous species^59^. Nevertheless, we recognize that traps are imperfect tools because they do not recover several species commonly found on human carcasses, including late colonizers, or because the communities sampled do not represent the typical insect fauna found on human carcasses^23^. Given the above, the fundamental issue becomes why baited traps are a poor proxy for predicting carcass colonization. A first element of explanation could lie in the fact that oviposition monitoring was carried out in two different years, replicating all four seasons, whereas the calibration and validation subsets were collected in a single year, covering only the insect active period from April to October. As a result, the calibration and validation data fail to encompass all natural variation, thus limiting our capacity to extrapolate the results to small cadavers (here: mouse) or human remains found in the same season but in a different year^58,60^. Nevertheless, it is common practice in forensic science to collect data in one year and use it to predict events occurring in another year, implying that our model conforms to current standards in the field. A second element of explanation could lie in the selection of dependent and independent variables. For example, we used the activity of adult females as response variable without considering additional information such as the age of the flies, the embryogenic status, i.e. gravid or not, the reproductive output of a single fly, or the population density in the area of interest. Including those parameters in prediction models could increase their accuracy as has already been shown in the control of sheep blow fly strike^29,37^. Models using not only the activity of gravid blow flies but including information on their reproductive output, such as how many eggs are laid by a single female, should be tested in future studies. In addition, we used abiotic variables recovered from local weather stations rather than measured at each individual site. As each site has its own microclimate in terms of solar radiation, vegetation, human influence, etc., it is unlikely that weather stations fully reflect microclimatic variations^61^. To improve species activity models, it is important to collect climate data at finer scales because the exposure of insects to environmental conditions is determined by small-scale local conditions, which can affect the biology and ecology of species^62^. Furthermore, there is strong evidence that many other factors such as biotic interactions and competition play an important part in determining species activity and oviposition^63^ and that non-climatic factors may be highly relevant in that sense^64^. Before including these variables in prediction models, we need to better understand the dynamics between necrophagous species in traps and on carcasses^65^. We suspect that the threshold for initiating flight is much lower than the decision to lay eggs, which has far-reaching consequences for the life history of female insects’ offspring^47^, and that a different set of influential parameters probably regulates each activity. A final element of explanation could be linked to what was said above about the need by *L. sericata* for fresh carrion as a source of protein for the maturation of the ovaries, and that female visits to animal necromass are not just for egg-laying but also for feeding.

To enhance the accuracy and reliability of forensic entomology, it is imperative to deepen our understanding of carrion ecology and the behavior of necrophagous insects. Monitoring studies employing baited traps and machine learning algorithm such as the random forests used here, could serve as invaluable tools. When it comes to prediction models to estimate the probability of carcass colonization, it is important to identify what information is pertinent and necessary for forensic investigation and its use in entomological casework. We assume that it is more critical to ascertain whether a species of interest will oviposit on a carcass and under which environmental circumstances (i.e., presence-absence), rather than quantifying how many specimens of each species will feed on a carcass. The interpretation of an estimated PMI_min_ is improved by knowing why or why not a particular fly species colonized a cadaver soon after death, rather than the extent of colonization. Therefore, we advocate for a focus on qualitative presence-absence models rather than quantitative ones. Along with all the other recommendations we make in this study, a pivotal objective should be to predict the colonization of human cadavers or, conversely, to improve our understanding of why a corpse is NOT colonized.

## Materials and methods

### Blow fly and environmental data

The calibration and validation subsets comprised presence/absence and occurrence data on the flight activity of the necrophagous blow flies *C. vicina*, *L. ampullacea*, *L. caesar* and *L. sericata* sampled on 152 days at five urban sites in the area of Frankfurt from April to October 2017. The flies were sampled using modified Red Top ^®^ Flycatchers (3 l, Ashmoat Ltd., Suffolk, UK) baited with 60 g of mashed chicken liver and were changed every 24 h. For a detailed description of the methodology, see Lutz et al.^15^. Thirty-four thousand six hundred thirty-seven female specimens were sampled. The test dataset included the oviposition activity of these four blow fly species on thawed mouse carcasses weighing 20–30 g, which were placed and sampled over a period of 240 days (10 days per month for 2 years) at five urban sites in the area Frankfurt from April 2019 until March 2021. They were surplus mice (*Mus musculus musculus*) from the breeding of the Central Research Facility of the Department of Medicine of the University Hospital, Frankfurt. Most of them were unusable siblings of mouse lines of different back- ground strains. On each sampling day, carcasses were placed on the ground between 6 and 7 AM and collected at sunset (+ 1 h). The carcasses were then transferred to ventilated plastic buckets and stored at room temperature until the blow fly larvae reached the post-feeding stage. At this point, the larvae were killed with near-boiling water and preserved in 96% ethanol until identified. By this, 218,111 larvae of the four species were sampled. For a detailed description of the methodology, see Lutz et al.^4^. Abiotic parameters recorded for each sampling day were available for both sampling periods. The parameters used for the models were: mean day temperature (°C), precipitation (mm), wind speed (km h^− 1^), barometric pressure (hPa), relative humidity (%), number of hours of sunshine for each day (h) and the day of the year. These data came from three local weather stations since none of the weather stations recorded all the required parameters. The weather station “Frankfurt-Höchst” was 8.8 km westwards of the sampling sites; “Offenbach-Wetterpark” was 8.52 km east of the sampling sites; and “Frankfurt-Westend” was 2.42 km north of the sampling sites.For the calculation of the parameters, hourly values during daylight were used. For detailed information on the calculation of these parameters, see Lutz et al.^4,15^.

### Statistical analyses

Predictor redundancy was reduced by using two different criteria, the bivariate r-squared and the variance inflation factor. Five predictors were retained, namely day of year, mean temperature, precipitation amount, wind speed, and barometric pressure. We randomly divided the 760 rows of the baited trap dataset into two sets: a calibration subset (608 rows; 80% of the dataset) and a validation subset (152 rows; 20% of the dataset). Subsequently, for each species, three different analysis methods were applied using the calibration subset: a Random Forest (RF) on abundance data, a Spatial RF on abundance data, and a RF on presence/absence data. RFs were developed using the rf function in the spatialRF package^66^ while spatial RFs were developed using the rf_spatial function of the same package in R^67^. The ntree parameter was set to 500 and the mtry was set to 2. Spatial autocorrelation was examined using Moran’s I. Random Forest being a stochastic algorithm that gives slightly different results on each run, we used the rf_repeat function to iterate the model 30 times and thus obtain the importance scores and response curves of the predictors for each run. Lastly, the RF models were validated using the validation subset and tested using the carcass set, as the spatial models cannot be extrapolated to a separate set due to the low transferability of spatial predictors^66^.

## Data Availability

The datasets analysed during the current study are available in the Mendeley Data repository, DOI: 10.17632/h76dd6twf6.1, https://data.mendeley.com/datasets/h76dd6twf6/1.
